# Cetacean Habitat Use and Occurrence in Fort-de-France Bay (Martinique)

**DOI:** 10.3390/ani15182640

**Published:** 2025-09-09

**Authors:** Coline Violo, Anatole Gros-Martial, Célia Ortolé, Marion Poupard, Morjane Safi, Benjamin de Montgolfier

**Affiliations:** 1 Aquasearch, ZAC Les Coteaux, Sainte-Luce 97228, Martinique; coline.violo@gmail.com (C.V.); grosmartial.anatole@gmail.com (A.G.-M.); c.ortole@aquasearch.fr (C.O.); m.poupard@aquasearch.fr (M.P.); m.safi@aquasearch.fr (M.S.); 2 Institut des Sciences de la Mer, Université du Québec à Rimouski, 310 Allées des Ursulines, Rimouski, QC G5L 2Z9, Canada; 3 Bio-Laurentia Aqua-Experts, 93 Route Melchior Poirier, St-Anaclet, QC G0K 1H0, Canada

**Keywords:** cetaceans, habitat use, behavioral patterns, anthropogenic disturbances, marine traffic, Fort-de-France Bay, spatial distribution, conservation strategies

## Abstract

**Simple Summary:**

Whales and dolphins are regularly seen near the coast of Martinique, particularly in the Bay of Fort-de-France. However, cargo ships, ferries, and other human activities also use this area, which may disturb these animals. The goal of this study was to understand how certain cetacean species use the bay over time and space and how their group size and behavior vary depending on location, sea depth and human activities. Data were collected through regular boat surveys over several years. The results show that some species are present year-round, while others appear only occasionally. Dolphins were observed more frequently in shallow waters close to the coast and often traveled or socialized in groups of various sizes. These findings suggest that the bay is an important area for several species. However, human activities may increase the risk of disturbance or collisions. This study underscores the importance of managing the shared use of the bay in a way that allows both economic activities and wildlife to coexist. These results will help local authorities and conservation groups make informed decisions to protect marine mammals while ensuring the area’s sustainable development.

**Abstract:**

While coastal species have been widely studied, active port areas in tropical island regions with intense maritime traffic remain critical, but habitats for cetaceans within the Caribbean have not been thoroughly studied. This study examines the spatial and temporal patterns of habitat use and the characteristics of groups of six cetacean species in the Bay of Fort-de-France in Martinique, an area with heavy marine traffic. Data were collected from 2018 to 2022 through systematic boat-based surveys. We analyzed standardized observations of group occurrence, size, behavior, and depth preference across different subzones of the bay. Our results reveal that *Stenella attenuata* and *Stenella longirostris* are the most frequently observed species, exhibiting distinct seasonal patterns, while other species occur more sporadically. Group sizes and behavioral patterns vary significantly across zones and depths. Larger, more interactive groups are generally observed in shallow areas. Several species’ preference for nearshore waters highlights the ecological value of the bay and the potential risks posed by anthropogenic pressures, such as noise, collisions, and habitat degradation. Our findings underscore the importance of considering cetacean habitat use in port management strategies. This study provides essential baseline knowledge to support conservation efforts and the development of mitigation measures that reconcile economic activities with the protection of marine biodiversity.

## 1. Introduction

Studying how cetaceans interact with their habitats and exhibit specific behaviors provides essential insights into their ecological roles and how they respond to environmental changes. These analyses are crucial for identifying key habitats, understanding species-specific needs, and ensuring the effective management of marine ecosystems [1]. Focusing on how cetaceans utilize their environments allows researchers to uncover critical patterns for conserving the species and their ecosystems [2]. The habitat use of cetaceans varies significantly across species and families, reflecting their diverse ecological niches [3]. For example, sperm whales (*Physeter macrocephalus*) dive to great depths to feed on squid, adapting to mesopelagic zones [4]. Coastal dolphins, such as bottlenose dolphins (*Tursiops truncatus*), form resident populations near shorelines and rely on shallow-water habitats for foraging and socializing [5]. Baleen whales, including humpback whales (*Megaptera novaeangliae*), undertake extensive migrations between feeding and breeding grounds, displaying intricate seasonal patterns of habitat utilization [6]. Understanding these behaviors and habitat preferences is imperative for identifying the environmental conditions necessary for cetacean survival and resilience. However, these ecological requirements are threatened by habitat degradation, climate change, and human activities [7,8,9]. Therefore, habitat use studies play a crucial role in identifying key ecological areas, assessing threats, and developing targeted conservation strategies [10]. By analyzing habitat preferences, researchers can determine the conditions necessary for cetacean growth and survival, thereby guiding conservation strategies to protect these vital habitats [11].

These efforts support cetacean populations and enhance the resilience of marine ecosystems. Research on habitat use is especially valuable in ecosystems affected by human activities, as it helps prioritize conservation efforts and inform strategies to mitigate human impacts [12]. Understanding habitat use and behavior is essential for cetacean conservation; however, these efforts face growing challenges from anthropogenic pressures, particularly in coastal and port areas. In these environments, where human activities are intense, it is crucial to understand how cetaceans adapt to ecological disruptions to ensure their survival. Port activities compromise cetacean health and survival by inducing various disturbances [13]. For example, acoustic disturbances from shipping, industrial operations, and construction interfere with cetacean communication and echolocation, resulting in stress, altered movement patterns, and reduced foraging efficiency [14].

Studies of habitat use offer practical solutions. For instance, one comprehensive review synthesized numerous studies demonstrating how research can inform strategies to reduce underwater noise from commercial shipping and mitigate its impact on critical cetacean habitats [15]. Behavioral disruptions, particularly those affecting foraging and resting, can have severe consequences. Chronic disturbances in feeding areas may force cetaceans to relocate to less optimal habitats, reducing their energy intake and negatively impacting their long-term fitness [16]. Interruptions to resting behaviors, especially in nursery habitats, interfere with energy conservation and increase stress levels, which has significant implications for mothers and calves [17]. Research into habitat use is essential for identifying and protecting these critical areas. Studies have highlighted the importance of preserving such habitats to maintain natural behaviors and ensure population stability over time. Chemical and plastic pollution from ports poses an additional threat to cetaceans. Runoff from industrial activities and plastic debris accumulates in cetacean tissues, leading to reproductive issues, weakened immune systems, and physical harm from ingestion [18]. Research emphasizes the importance of habitat-use studies in assessing exposure to persistent pollutants and in shaping targeted conservation strategies [19]. Furthermore, the physical presence of vessels in busy port areas increases the risk of collisions, posing a direct threat to the well-being of cetaceans [20].

The variety of human-caused threats to cetaceans highlights the need for thorough habitat use studies to address these challenges. By identifying and mitigating these impacts, conservation initiatives can protect cetaceans and enhance the resilience of marine ecosystems and biodiversity [11,12]. Although the impact of human activities on cetaceans is well-known, it is especially severe in bays and port areas, creating a paradox. These habitats, which are crucial for foraging, resting, and protection, are also high-risk sites. Understanding the importance of bays and port areas for cetaceans is essential, as they are crucial sites due to a combination of ecological and environmental factors. These areas are especially significant because they support abundant prey species, such as fish and squid, which thrive in nutrient-rich waters, often fueled by upwelling or freshwater inflow [21]. This results in feeding grounds with high primary productivity, making them ideal for cetaceans [22]. Furthermore, bays offer shelter from strong ocean currents and predators, providing cetaceans with safe environments to rest, socialize, and engage in breeding activities [22]. The calmer waters of these areas are particularly valuable for mothers with young, offering protection and access to vital resources [23]. While these ecological advantages attract cetaceans to bays and port areas, these habitats are not without significant risks. The features that provide a favorable environment for cetaceans often coincide with areas of intense human activity, including shipping, industrial operations, and recreational boating [24].

Consequently, cetaceans in these areas face multiple anthropogenic threats, including acoustic disturbances, chemical pollution, physical disruptions, and collisions with vessels. This creates a paradox: while these areas offer ecological benefits that attract cetaceans, they also expose them to significant risks that can compromise their health and survival [9]. This phenomenon underscores the necessity of rigorous research to develop conservation strategies that preserve the ecological value of these habitats and mitigate the harmful effects of human activities. Studies have shown that management approaches designed to reduce anthropogenic impacts can protect cetacean populations in these vulnerable areas [25]. For instance, research conducted in other bays around the world sheds light on the impact of such disturbances on cetaceans. In Banderas Bay, Mexico, for instance, research on dolphins revealed that port activities and tourism-related vessel traffic significantly altered their behavior and distribution. Specifically, noise pollution and physical disruptions led to changes in habitat use and reduced social interactions [26]. A similar phenomenon occurred in Algeciras Bay, Spain, where intensive port operations significantly altered cetacean behavior and distribution, particularly with regard to shipping noise [26]. Researchers found that cetaceans avoided high-traffic zones, highlighting the need for targeted mitigation measures [27]. These comparative studies underscore the importance of understanding the intricate relationship between cetaceans and human activities in port environments. They provide a valuable foundation for investigating comparable impacts in the Bay of Fort-de-France and for developing region-specific conservation strategies.

The Bay of Fort-de-France, located on the western coast of Martinique, is an important ecological area because of its rich marine biodiversity and proximity to Martinique’s Great Seaport. The bay is home to a variety of marine species, including several species of cetaceans that are frequently observed in its waters. The most commonly sighted species include the pantropical spotted dolphin (*Stenella attenuata*), the bottlenose dolphin (*Tursiops truncatus*), and the sperm whale (*Physeter macrocephalus*). Other species, such as the humpback whale (*Megaptera novaeangliae*), use the area for reproduction and rest [28]. The bay’s diverse habitats, including seagrass beds, coral reefs, and shallow coastal waters, provide vital ecological functions for cetaceans [28]. These habitats are crucial for foraging because nutrient-rich waters resulting from upwelling and freshwater inflows support an abundance of prey, including squid and fish [24].

The calm waters of the bay also serve as a refuge for mothers with calves, making it an important nursery habitat for cetaceans [25]. Despite its ecological significance, the Bay of Fort-de-France faces considerable challenges. The Great Seaport of Martinique, the largest commercial port in the Caribbean, introduces several anthropogenic pressures that impact the marine environment. Shipping traffic, industrial operations, and recreational boating generate noise pollution, water contamination, and physical disturbances that threaten cetacean health and survival. The bay’s ecological appeal to cetaceans—abundant prey and sheltered waters—conflicts with human activities. This creates a paradox where cetaceans are drawn to these habitats yet simultaneously exposed to stressors [9]. These disturbances can disrupt cetacean behavior, which could impact the long-term viability of local populations [15,29]. The Agoa Sanctuary, a marine protected area aimed at conserving cetacean species throughout the Caribbean, supports the protection of cetaceans in the region. The sanctuary legally protects several species and ensures that critical habitats within the Bay of Fort-de-France are preserved. However, given ongoing anthropogenic pressures, further research and monitoring are essential to develop effective management strategies and gain a comprehensive understanding of how cetaceans interact with port environments under human influence [30].

This study aims to analyze how cetaceans use Fort-de-France Bay to gain a deeper understanding of its ecological importance for these animals and the challenges they face due to human activities. The findings will contribute to updated knowledge of the diversity and frequency of observed cetaceans, as well as the development of targeted management strategies for marine conservation. These strategies will ensure the long-term survival of cetacean populations and the health of the marine ecosystem [31,32]. This study focuses on analyzing cetacean habitat use in Fort-de-France Bay, particularly near the Grand Port Maritime de la Martinique. Using a long-term dataset, the research will examine the spatial distribution and behavioral patterns of cetaceans in response to natural and anthropogenic factors. The objective is threefold: first, to evaluate the ecological function of these habitats; second, to understand the consequences of human activities; and third, to identify critical areas for conservation. Long-term monitoring programs are imperative for tracking changes in cetacean behavior and distribution over time, providing invaluable insights into the influence of human activities on marine species. Collating these data will support developing effective conservation strategies that balance the needs of cetaceans with the demands of port activities, ensuring their sustainable coexistence. The Grand Port Maritime de Martinique requested this study to better understand the impact of its activities on cetaceans and develop coexistence strategies.

## 2. Materials and Methods

### 2.1. Study Area

**Figure 1:**
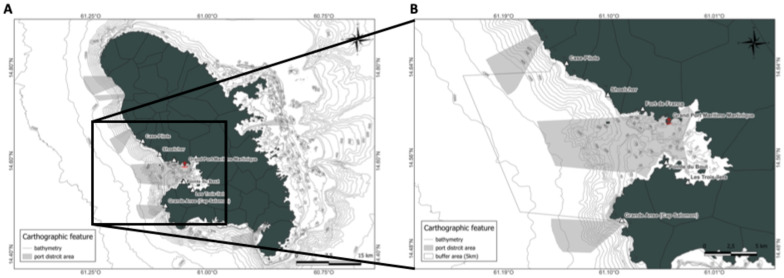
Location of the study area in Martinique (**A**) and the Great Seaport of Martinique in (**B**).

This study focuses on a specific area of Martinique, extending from Case-Pilote in the northwest to Cap Salomon in the southwest (Figure 1A). This area included the bottom of the Bay of Fort-de-France and an offshore section extending 18 nautical miles (33 km). It was defined by a 5-km buffer zone near the Great Seaport of Martinique (the island’s main commercial port), as well as the ports and marinas of the surrounding towns: Bourg des Trois-Îlets, Pointe du Bout, and Schoelcher. The total area covered by the zone was 390 km^2^ (Figure 1B).

### 2.2. Sampling Period

Ethological monitoring of cetaceans in the study area was conducted using two databases. The first is a historical database consisting of sightings collected by Aquasearch from 2013 to 2021. The second is a new database fed by sightings conducted as part of this project in 2022.

#### 2.2.1. Historical Database

From 2013 to 2021, the Aquasearch team and partner whale watchers maintained this database, which contained all cetacean sightings along the Caribbean coast. It comprised biological data on sightings, including species identified, number of individuals observed, group composition (calves, juveniles, adults), and animal behavior (socialization, movement, hunting, breeding and rest; Table 1). It also collected environmental data, including wind, sea state, visibility, and the presence of boats, birds, or debris. Finally, the date, day, and time of the sighting were recorded.

#### 2.2.2. Field Database

**Figure 2:**
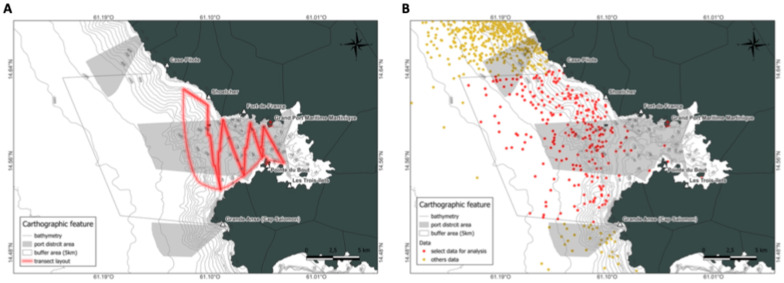
Transect monitoring route for updated database in (**A**) and Mapping of selected data for study area in (**B**).

As part of this project, a database was populated with observations made within the designated study area of the Bay of Fort-de-France, following a transect protocol. The Aquasearch team has been executing this protocol since 26 January 2022, at a frequency of twice per week for 12 months. Observations at sea generally took place in the morning. The team departed from the port of Le Bourg des Trois-Îlets aboard a 6-m Bombard Zodiac equipped with a 115-horsepower Mercury engine. The transects were sailed from east to west at a speed of 12 knots (Figure 2A), crossing the bay on seven routes for a total distance of approximately 40 km and ending in Case-Pilote. The primary objective of this approach was to ensure comprehensive coverage of the bay and maximize the probability of encountering cetaceans. Spotting was conducted through visual detection; the presence of flippers, blowholes, or hunting birds indicated potential schools of fish, and so potential cetaceans. During the sighting, the number of individuals, their behavior, and activities were noted. When cetaceans were sighted, the vessel’s speed was reduced in accordance with the “Charte d’approche du Sanctuaire Agoa,” [33] and a distance of 100 m was maintained to avoid disturbing the animals. The study area included not only the transects but also cetaceans observed during the routes and those present in the vicinity of the bay, as derived from historical data. Only sightings reported by trained whale watchers collaborating with the Aquasearch team, and using a standardized observation protocol, were included to ensure data consistency.

### 2.3. Data Collection

The analysis of sightings combined data from 2022 with data from the historical database. This approach updated the 2022 cetacean sightings, resulting in 1417 observations.

Using QGIS Desktop 3.22.0, a total of over 1417 initial observations were filtered to retain only those located within the designated study area, which includes both the buffer zone and the catchment area. After applying this spatial selection, 301 relevant observations were retained for analysis (Figure 2B). These observations were then used to compare those in the buffer zone (also known as the adjacent zone) with those in the catchment area of the Great Seaport of Martinique. This comparison aimed to study the ecology and behavior of different species in their environment and the impact of human activity.

### 2.4. Statistical Analysis

A global approach was adopted for the statistical analysis, whereby the proportion of observations per year and per month was calculated for all species combined. The ratio of individuals to observations was also examined, which highlighted the importance of standardizing the data for subsequent analyses. Then, the analysis was performed individually for the six species with the highest number of observations. For each species, the number of observations per year and month was assessed, as well as group size. Group size was standardized by year and month by dividing the number of individuals by the number of observations. Group size was also standardized by depth. To explore the relationship between cetacean behavior and bathymetry, a heatmap-type correlation plot was generated. Observations were grouped by behavioral category, bathymetric class (depth intervals), and area. A standardized density of individuals per observation was calculated and visualized using a tile plot (geom_tile) in R 4.3.0 with the ggplot2 package [34]. Depth values were classified into eight intervals ranging from <−1500 m to >−50 m. Finally, to complete the mapping study, the proportion of newborn and behavioral observations was estimated. All graphs were generated using RStudio 2025.05.1+513 [34].

### 2.5. Spatial Analysis

A spatial analysis was conducted using a specific version of QGIS software (3.40.10 version). A density map was created to illustrate the concentration of observations made during fieldwork. The map used a grid, and each cell was assigned a value proportional to the number of individuals observed. This enabled the identification of areas of high abundance. A similar map was created to visualize the density of newborns. Finally, a map showing the locations where the species of interest exhibited behaviors was produced.

## 3. Results

### 3.1. Stenella attenuata

**Figure 3:**
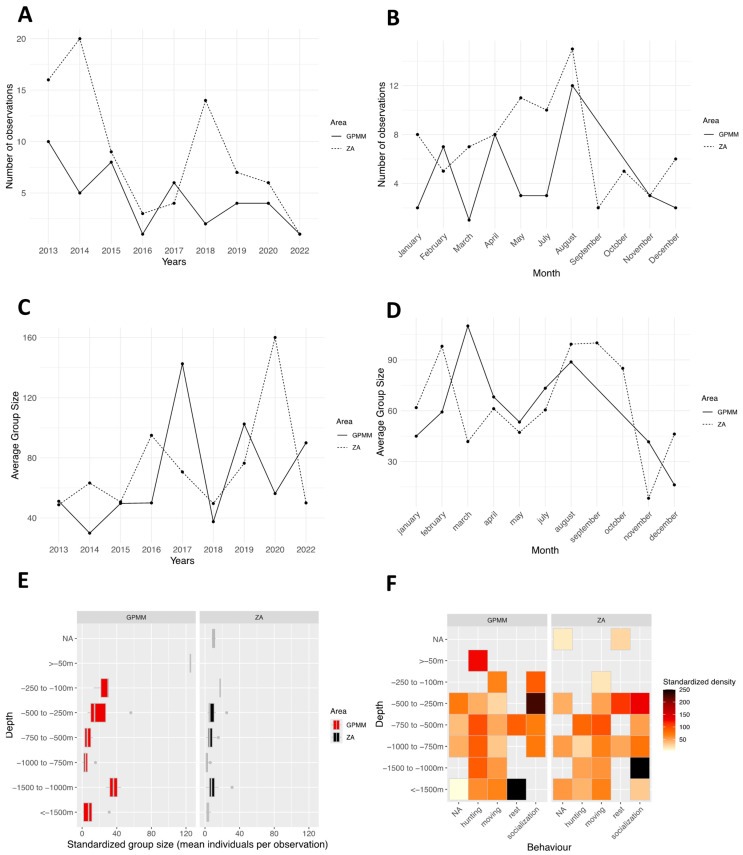
Distribution of *Stenella attenuata* sightings by month (**A**) and year (**B**), the average group size by month (**C**) and year (**D**), the distribution of sightings standardized by depth (**E**), and the density of sightings according to behavior and area by depth (**F**) (ZA: adjacent area; GPMM: Grand Port Maritime de Martinique).

From 2013 to 2022 (excluding 2021, during which no individuals were recorded in the area due to the very limited number of field surveys caused by the COVID-19 pandemic), the species was recorded annually in both Grand Port Maritime de Martinique (GPMM) and the adjacent area (ZA) (Figure 3A). The frequency of observations ranged from two to 15 per year. Monthly distribution of observations indicates a presence throughout the year, except in June (Figure 3B). The absence of observations in June may be partly explained by the seasonal decline in marine-based tourism activities in Martinique during this period, resulting in fewer whale-watching trips and reduced field effort. Observation counts vary by month and area. In the GPMM, they range from 1 to 12, and in the adjacent area, they range from 2 to 15, with peaks in August for both areas and in May and July for the adjacent area. Standardized group sizes by year and study area ranges from 20 to 140 individuals in the GPMM and from 50 to 160 individuals in the adjacent area. (Figure 3C) An alternating pattern is observed: years in which larger groups are recorded in one area, while smaller groups dominate the other. Additionally, from 2016 to 2022, group sizes appear larger (40 to 160 individuals) than during the 2013–2016 period (20 to 60 individuals). Monthly variations in group sizes range from 40 to 120 individuals are primarily observed from January to April and from August to October (Figure 3D). Smaller groups ranging from 10 to 45 individuals are recorded from November to December. Distribution of group sizes by depth in the GPMM shows that larger groups are observed at depths between 100 and 250 m and between 1000 and 1500 m (Figure 3E). In the adjacent area, groups tend to be smaller and are distributed across a wider range of depths. There are fewer occurrences of large groups concentrated at specific depths. This dynamic is reflected in monthly variations as well. Groups of 40 to 120 individuals are mainly observed between January and April and between August and October. In contrast, from November to December, group sizes appear smaller, with a maximum of 10 to 45 individuals. Behavioral densities by depth in the GPMM, present high densities of socializing individuals occur between 250 and 500 m, and resting behavior is mainly recorded at depths greater than 1500 m (Figure 3F). Foraging behavior is concentrated in shallow waters (less than 50 m). In the adjacent area, socializing occurs between 1000 and 1500 m, resting occurs between 250 and 500 m and 750 and 1000 m, and other behaviors are more evenly distributed with depth.

**Figure 4:**
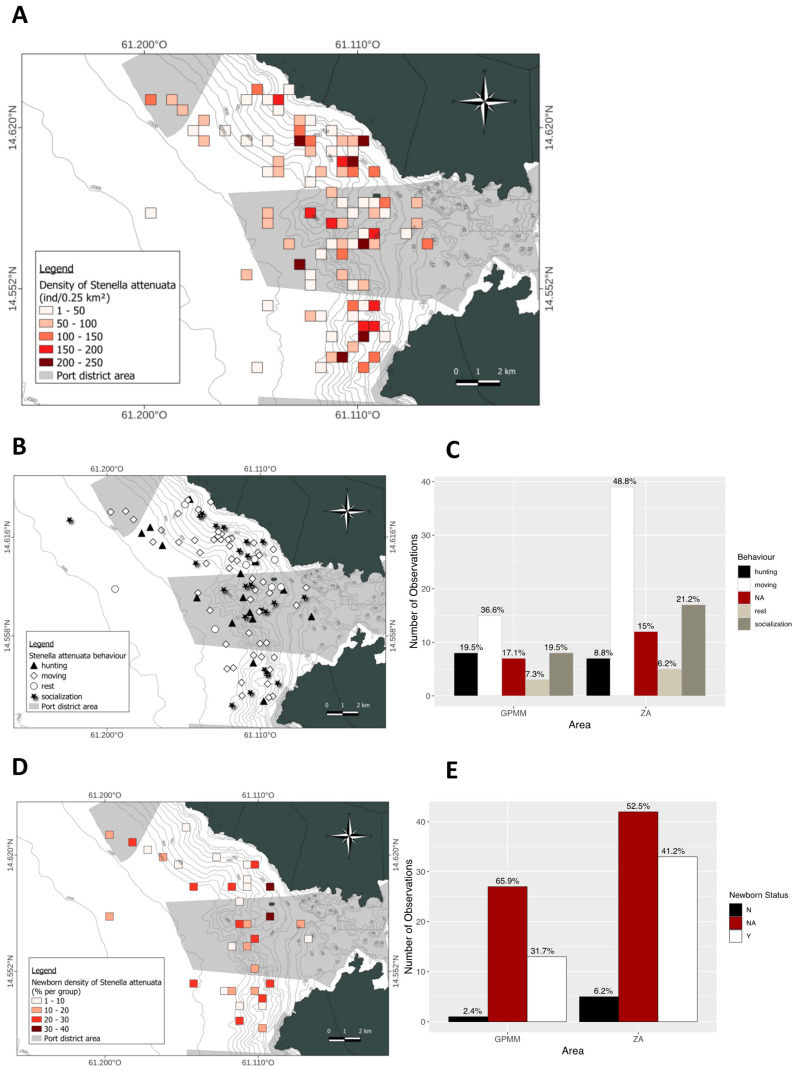
Mapping of *Stenella attenuata* individual density (**A**), density of juveniles observed (**B**) and distribution by area (**C**), distribution of behavior observed (**D**) and distribution by area (**E**) (ZA: adjacent area; GPMM: Grand Port Maritime de Martinique), (N: any newborn, Y: observation of newborn, NA: no data).

Density of *Stenella attenuata* individuals per 0.25 km^2^ (Figure 4A). The spatial distribution of these densities is heterogeneous within each area. The highest densities are found in the deep waters of the GPMM. In the adjacent area, however, the densities are concentrated near the coast. The species is rarely present in the GPMM (Figure 4A). Regarding group composition, the presence of juveniles varies between areas. The distribution of groups containing juveniles is heterogeneous; some groups have a high juvenile density (30–40% of the group) in both the GPMM and near the coast. However, the proportion of groups containing juveniles is higher in the adjacent area (41%) than in the GPMM (31%) (Figure 4B,C). Finally, behavioral distribution and proportions differ between study areas. In both areas, traveling is the most frequently observed behavior at all depths. In the GPMM, foraging and socializing occur at similar rates, and these behaviors are observed up to the entrance of Fort-de-France Bay. In the adjacent area, socializing is the second most frequent behavior, mainly occurring near the coast. This is followed by foraging and resting (Figure 4D,E).

### 3.2. Tursiops truncatus

**Figure 5:**
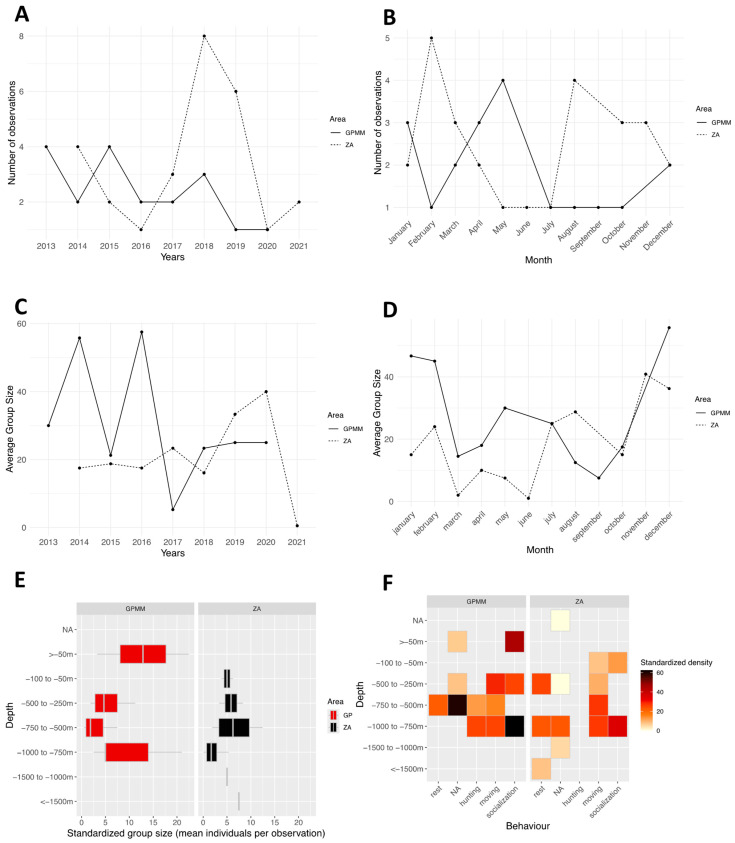
Distribution of *Tursiops truncatus* sightings by month (**A**) and year (**B**), the average group size by month (**C**) and year (**D**), the distribution of sightings standardized by depth (**E**), and the density of sightings according to behavior and area by depth (**F**) (ZA: adjacent area; GPMM: Grand Port Maritime de Martinique).

Standardized number of observations per year and zone for Bottlenose dolphin range from 1 to 8 between 2013 and 2021 (Figure 5A). Peaks of eight and six observations were recorded in 2018 and 2019, respectively, in the adjacent zone, with limited variation in other years. The monthly distribution (Figure 5B) shows that the species was observed every month, with one to five records per month. In the GPMM, higher occurrences were noted in January, April, and May, while in the adjacent zone, they occurred in February, August, and October. Group sizes by year and zone (Figure 5C) were generally larger in the GPMM (15–55 individuals) than in the adjacent zone (1–40 individuals), with contrasting annual trends between zones. Monthly group sizes (Figure 5D) followed a similar trend in both zones, ranging from 35 to 120 individuals from January to October and decreasing to 10–45 individuals in November and December. Group size by depth (Figure 5E) was relatively similar across zones, with higher variability between 750 and 1000 m. In the GPMM, groups of five to ten individuals were also observed between 250 and 750 m. In the adjacent zone, group size variability was lower at these depths, increasing at greater depths. Density by depth and behavior, in the GPMM, high densities during socializing were recorded at 750–1000 m and <50 m; resting occurred mainly at 500–750 m; and traveling and foraging were observed at 250–1000 m (Figure 5F). In the adjacent zone, socializing and traveling occurred at similar depths; however, no foraging was recorded. Resting individuals were mostly observed between 250 and 500 m.

**Figure 6:**
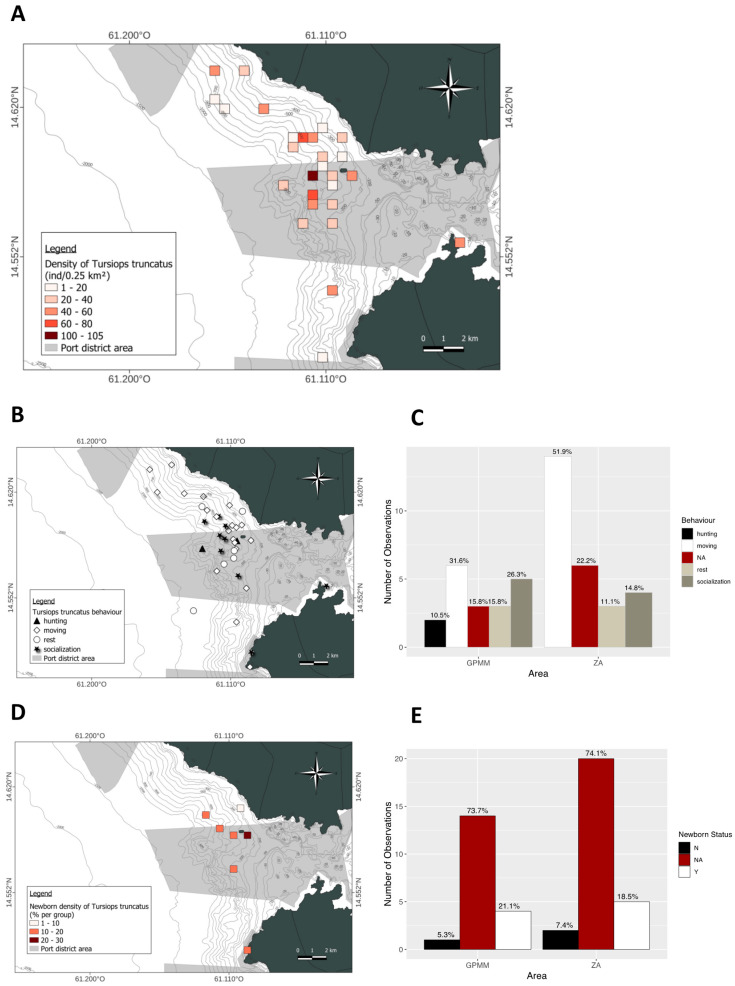
Mapping of *Tursiops truncatus* individual density (**A**), density of juveniles observed (**B**) and distribution by area (**C**), distribution of behavior observed (**D**) and distribution by area (**E**) (ZA: adjacent area; GPMM: Grand Port Maritime de Martinique), (N: any newborn, Y: observation of newborn, NA: no data).

Spatial density is higher north of the GPMM (at depths of 200–800 m) and in the northern part of the adjacent zone (Figure 6A). Two groups were recorded south of the GPMM, and one was recorded inside the Bay of Fort-de-France. The presence of juvenile groups was more prevalent in the GPMM (21%) than in the adjacent zone (18.5%) (Figure 6B,C). Most groups contained 10–20% juveniles; one group in the GPMM contained 20–30%. Behavioral observations in the GPMM, showed that traveling was the most frequent behavior (31.6%), followed by socializing (26.3%), resting (15.8%), and foraging (10.5%) (Figure 6D,E). In the adjacent zone, traveling was the most common behavior (51.9%), followed by socializing (14.8%) and resting (11.1%). Foraging was not recorded. Socializing and foraging were concentrated in the northern GPMM, while traveling and resting were more broadly distributed across depths and locations.

### 3.3. Physeter macrocephalus

**Figure 7:**
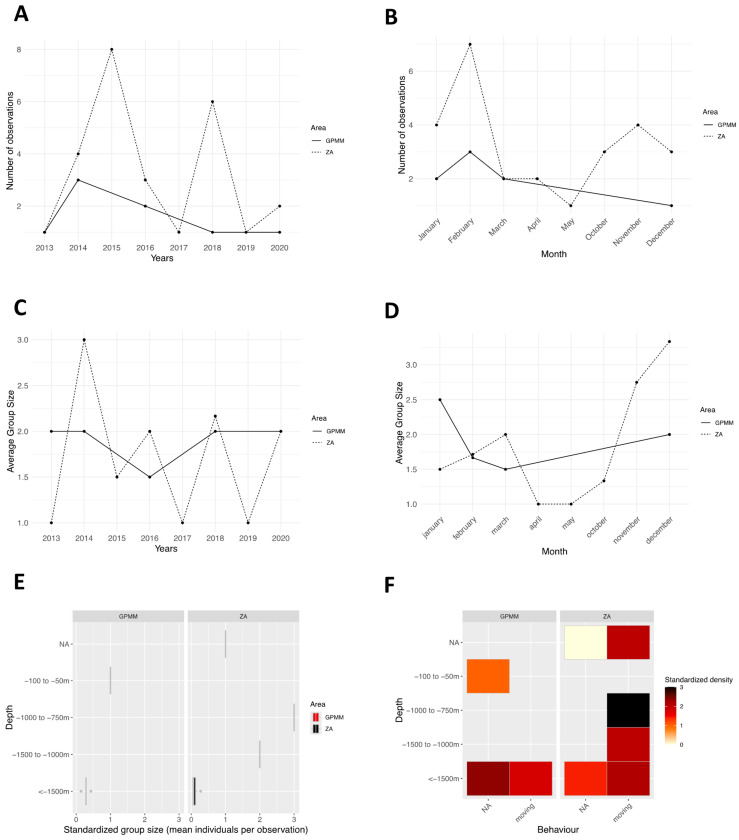
Distribution of *Physeter macrocephalus* by month (**A**) and year (**B**), the average group size by month (**C**) and year (**D**), the distribution of sightings standardized by depth (**E**), and the density of sightings according to behavior and area by depth (**F**) (ZA: adjacent area; GPMM: Grand Port Maritime de Martinique).

The number of observations per year and per zone ranged from one to three in the GPMM zone and from one to eight in the adjacent zone (Figure 7A). Two peaks were recorded in the adjacent zone in 2015 and 2018, with more than six observations. Monthly variations (Figure 7B) showed sightings every month except in June, July, and August. In the GPMM zone, the number of sightings ranged from one to three, while in the adjacent zone, it reached seven, with higher values recorded in January, February, and from October to December. Standardized group size by year (Figure 7C) remained stable in the GPMM zone (between one and two individuals) and varied slightly in the adjacent zone (between one and three individuals). There were opposite trends between zones over the years. Monthly variations in group size (Figure 7D) displayed contrasting patterns between zones with no consistent temporal trend. Group distribution by depth and zone (Figure 7E) revealed that, in the GPMM zone, individuals were mostly observed between 50 and 100 m and beyond 1500 m, whereas, in the adjacent zone, sightings occurred mainly between 750 and 1500 m.

**Figure 8:**
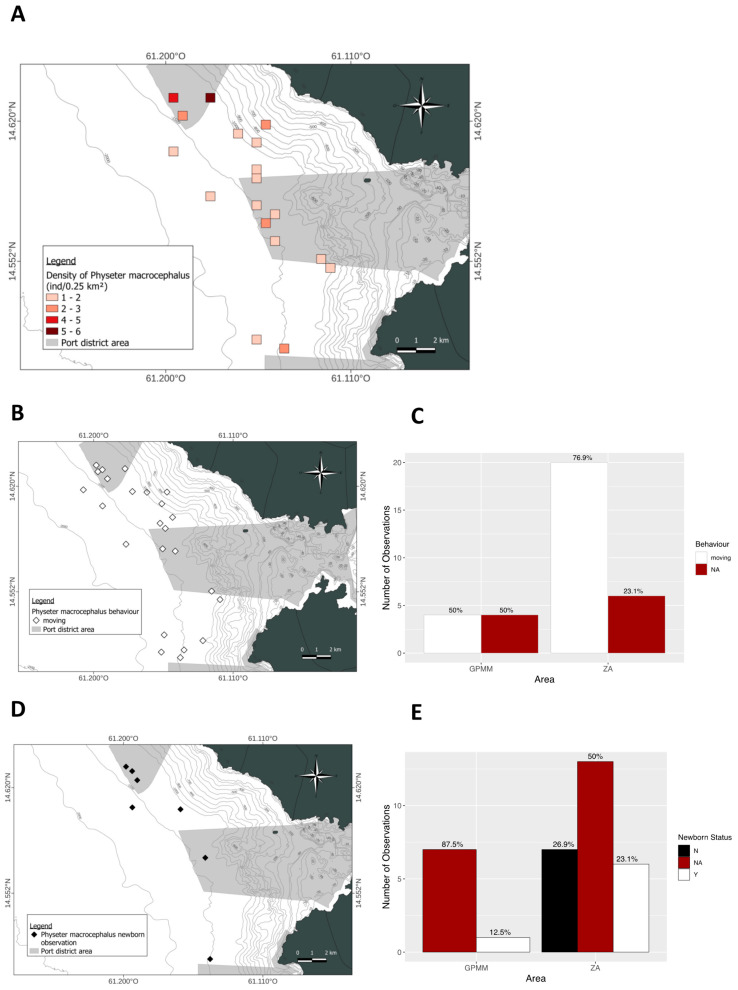
Mapping of *Physeter macrocephalus* individual density (**A**), density of juveniles observed (**B**) and distribution by area (**C**), distribution of behavior observed (**D**) and distribution by area (**E**) (ZA: adjacent area; GPMM: Grand Port Maritime de Martinique), (N: any newborn, Y: observation of newborn, NA: no data).

Regarding behavior (Figure 7F), only travel was documented. Individuals were observed at 1500 m in the GPMM zone and between 750 and 1500 m in the adjacent zone. Figure 8A illustrates the spatial density of sperm whale sightings. Individuals were observed offshore in the GPMM zone and along the coast in the adjacent zone. They were primarily observed between 800 and 2000 m deep. One sighting was recorded at the entrance of the Bay of Fort-de-France at a depth of 50 m. Concerning the presence of juveniles (Figure 8B,C), 12.5% and 23.1% of individuals were observed with calves in the GPMM and adjacent zones, respectively. Most of these sightings occurred in offshore areas deeper than 1000 m. Finally, in terms of behavior (Figure 8D,E), only traveling was observed.

### 3.4. Lagenodelphis hosei

**Figure 9:**
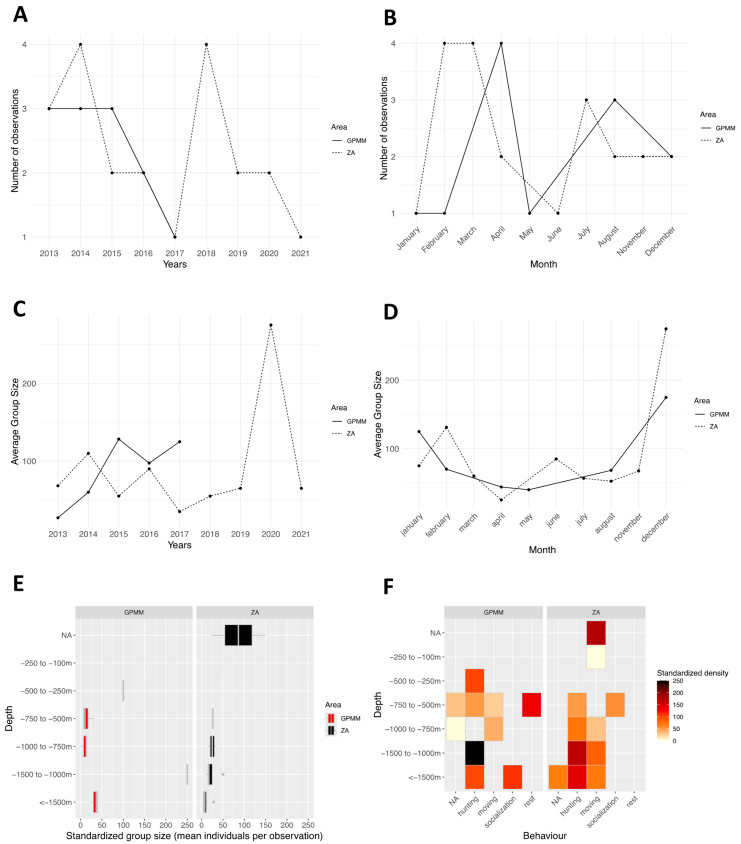
Distribution of *Lagenodelphis hosei* by month (**A**) and year (**B**), the average group size by month (**C**) and year (**D**), the distribution of sightings standardized by depth (**E**), and the density of sightings according to behavior and area by depth (**F**) (ZA: adjacent area; GPMM: Grand Port Maritime de Martinique).

Number of observations per year and zone reveal consistent sightings from 2013 to 2021 (Figure 9A). In the GPMM zone, one to three observations were recorded annually from 2013 to 2017. In the adjacent zone, the number of observations ranged from one to four, with at least one sighting every year. Monthly patterns indicate that the species was observed in all months except September and October (Figure 9B). Sightings ranged from one to four per month, with similar seasonal trends across both zones. Higher observation rates were recorded between February and April, as well as in July and August. Group sizes varied substantially from year to year (Figure 9C), ranging from 20 to 280 individuals. In the GPMM zone, group sizes ranged from 20 to 125 individuals. In the adjacent zone, group sizes ranged from 30 to 115 individuals. Exceptionally large aggregations exceeding 270 individuals were recorded in 2020. Monthly variations in standardized group size by zone reveal consistent group sizes between 25 and 125 individuals in the adjacent zone, except for a peak above 250 individuals in December (Figure 9D). In the GPMM zone, group sizes ranged from 40 to 170 individuals. In the GPMM zone, dolphins were predominantly observed in three depth ranges: 500–750 m, 750–1000 m, and >1500 m (Figure 9E). The average group size was stable at around 20–25 individuals in these ranges. A similar distribution was observed in the adjacent zone, with additional sightings occurring at depths between 1000 and 1500 m. Density of observations by behavior, depth, and zone, revealing the presence of all four behavioral categories in both zones (Figure 9F). In the GPMM zone, resting behavior was only observed at depths between 500 and 750 m. Socializing and foraging predominantly occurred beyond 1500 m, though foraging also occurred between 750 and 1000 m and occasionally between 250 and 750 m. Traveling behavior was recorded between 500 and 1000 m. In the adjacent zone, foraging behavior exhibited a similar distribution, occurring between 500 and >1500 m, with higher densities in deeper waters. Travel behavior was widespread, particularly between 750 and 1500 m, and socializing occurred only at 500–750 m. Resting behavior was not recorded.

**Figure 10:**
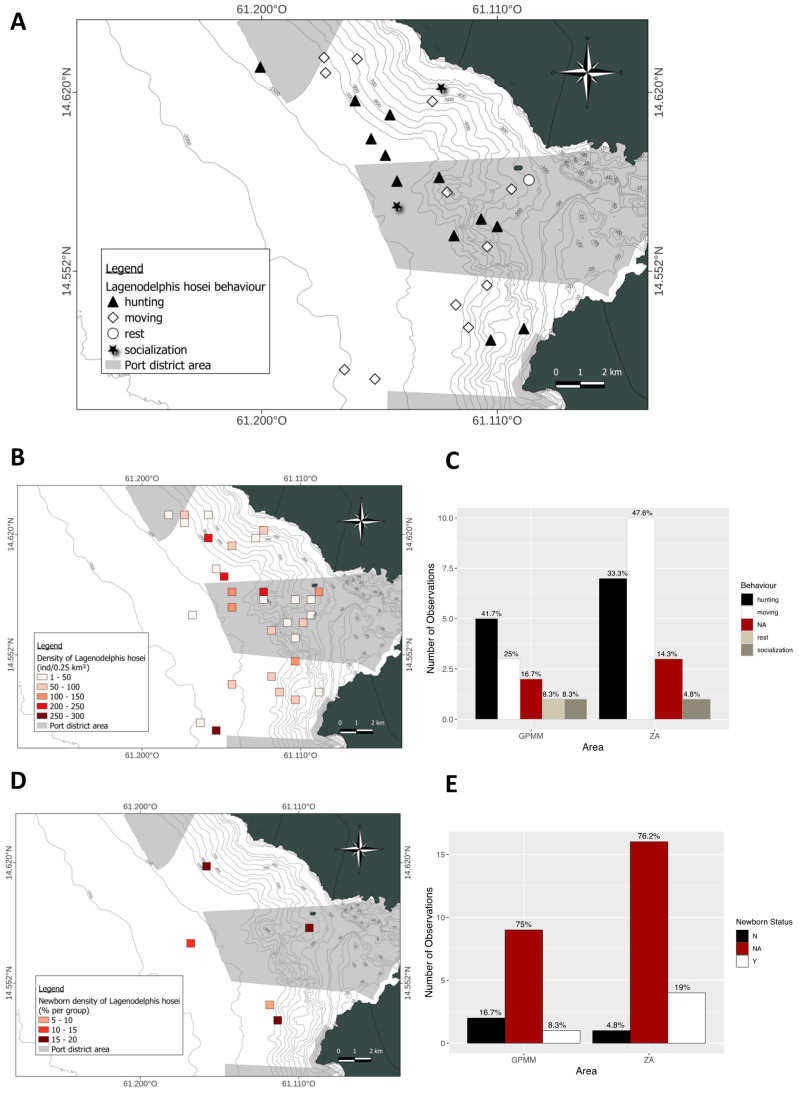
Mapping of *Lagenodelphis hosei*, individual density (**A**), density of juveniles observed (**B**) and distribution by area (**C**), distribution of behavior observed (**D**) and distribution by area (**E**). (ZA: adjacent area; GPMM: Grand Port Maritime de Martinique), (N: any newborn, Y: observation of newborn, NA: no data).

The spatial distribution of sightings throughout the study area exhibited a heterogeneous pattern (Figure 10A). Groups of various sizes were observed in both zones, ranging from the coastal areas to the offshore regions and extending from the northern to the southern parts of the GPMM. Regarding the presence of juveniles (Figure 10B,C), 8.3% of groups in the GPMM zone and 19% of groups in the adjacent zone included juveniles. These juveniles represented 5% to 20% of the composition of the groups and were mostly found in waters that were 800 m deep or greater, except for one sighting in 400-m-deep waters in the GPMM zone. The spatial distribution of behaviors (Figure 10D,E) revealed that foraging was the most prevalent behavior in the GPMM zone (41.7%), followed by traveling (25%). Socializing and resting accounted for 8.3% of observations each.

These behaviors occurred offshore at significant depths outside the Bay of Fort-de-France. In the adjacent zone, traveling dominated (47.6%), followed by foraging (33.3%) and socializing. No resting behavior was recorded in this zone. As in the GPMM zone, foraging and traveling behaviors were mostly observed offshore, while socializing took place closer to the coast.

### 3.5. Globicephala macrorhynchus

**Figure 11:**
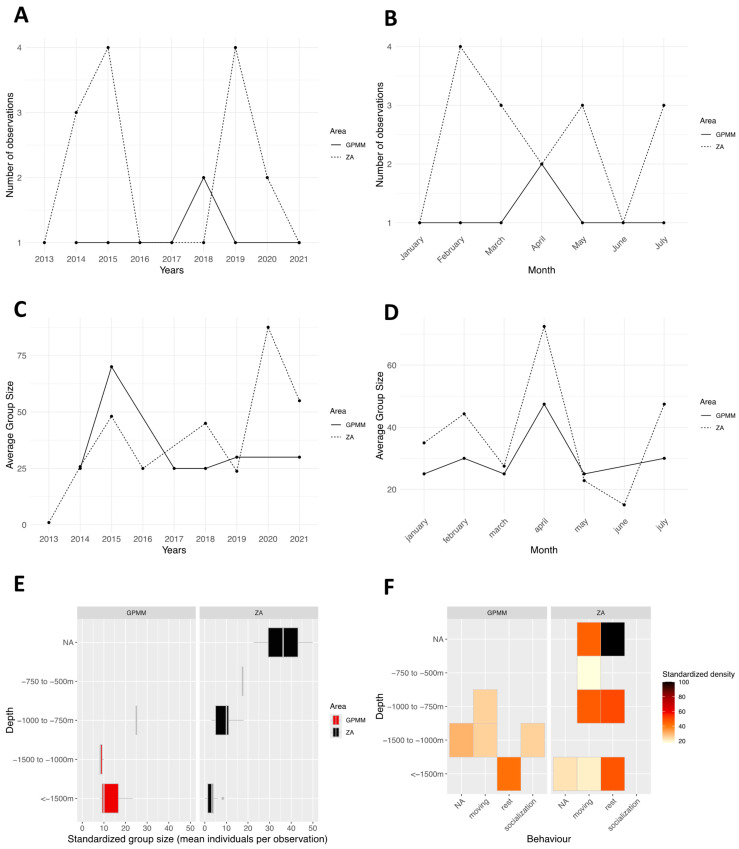
Distribution of *Globicephala macrorhynchus* by month (**A**) and year (**B**), the average group size by month (**C**) and year (**D**), the distribution of sightings standardized by depth (**E**), and the density of sightings according to behavior and area by depth (**F**) (ZA: adjacent area; GPMM: Grand Port Maritime de Martinique).

The number of standardized yearly observations ranged from one to two in the GPMM and from one to four in the adjacent zone between 2014 and 2021 (Figure 11A). Temporal trends differed between the two areas. Monthly observations (Figure 11B) occurred from January to July in both zones. In the GPMM, one sighting was recorded per month, except in April when two sightings occurred. In the adjacent zone, observations were more variable, peaking in February with four sightings, and fewer sightings occurring in other months. Group size by year (Figure 11C) ranged from 25 to 75 individuals in the GPMM zone and showed a decreasing trend over time. In contrast, group size in the adjacent zone ranged from one to 90 individuals, exceeding 70 individuals from 2020 onward. Monthly group size (Figure 11D) followed a similar pattern in both zones, ranging from 25 to 40 individuals from January to March, increasing to 45 to 75 individuals in April, and decreasing again to 10 to 45 individuals between May and July. Regarding depth (Figure 11E), group sizes in the GPMM zone ranged from 8 to 18 individuals at depths greater than 1500 m, with little variation between 1000 and 1500 m. In the adjacent zone, group size was more variable at a depth of 750–1000 m and more stable at depths greater than 1500 m. Density by depth and behavior in the GPMM zone (Figure 11F) demonstrated that traveling occurred primarily between 750 and 1500 m (with few individuals), resting occurred primarily above 1500 m, and socializing occurred primarily between 1000 and 1500 m. In the adjacent zone, traveling was the dominant behavior between 750 and 1000 m. Resting was also observed in this area. No social behavior was observed in this zone.

**Figure 12:**
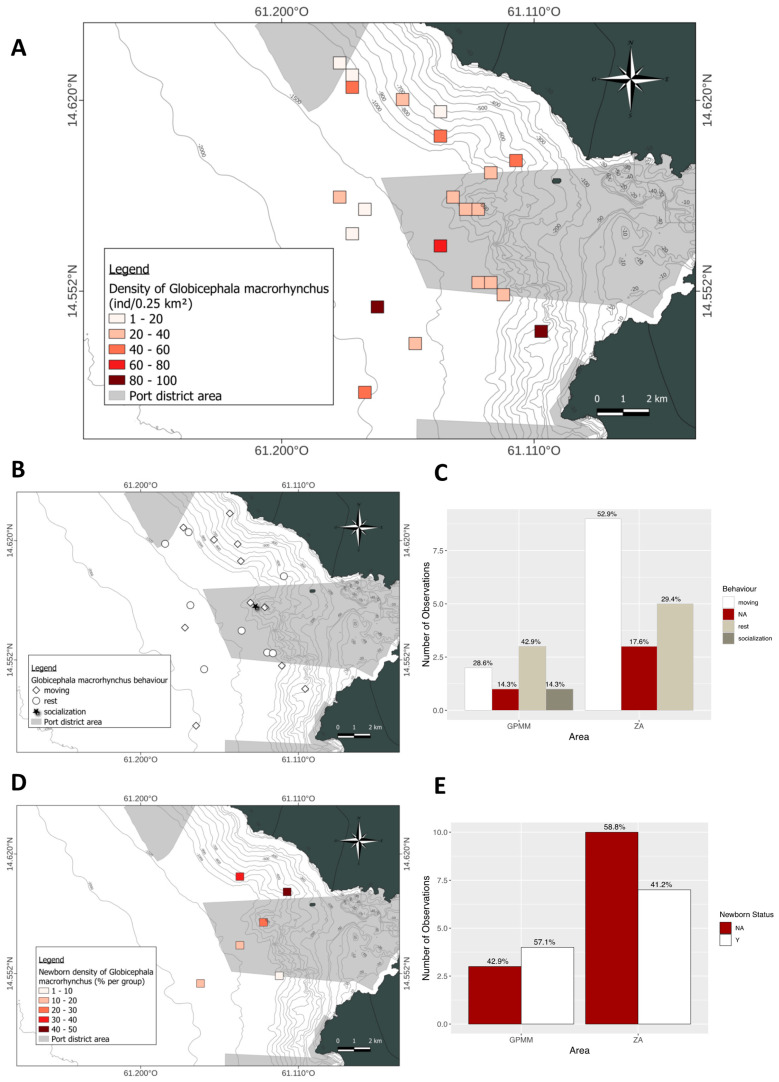
Mapping of *Globicephala macrorhynchus*, individual density (**A**), density of juveniles observed (**B**) and distribution by area (**C**), distribution of behavior observed (**D**) and distribution by area (**E**) (ZA: adjacent area; GPMM: Grand Port Maritime de Martinique), (N: any newborn, Y: observation of newborn, NA: no data).

In the GPMM zone, groups of 20–40 individuals were primarily found offshore in the Bay of Fort-de-France (Figure 12A). In the adjacent zone, group sizes ranged from one to 20 individuals, and some larger groups of 80–100 individuals were also recorded. Regarding the presence of juveniles (Figure 12B,C), 57.1% of GPMM sightings included juveniles compared to 41.2% in the adjacent zone. In the GPMM zone, juveniles made up 10–30% of the group composition; in the adjacent zone, however, their proportion was more variable (1–50%). Behavioral distribution (Figure 12D,E) differed between zones. In the GPMM zone, resting was the most frequent behavior (42.9%), followed by traveling (28.6%) and socializing (14.3%). In the adjacent zone, traveling predominated (52.9%), followed by resting (29.4%). No social or foraging behavior was recorded in this zone. Overall, behaviors were heterogeneously distributed across both deeper and shallower waters in both areas.

### 3.6. Megaptera novaeangliae

**Figure 13:**
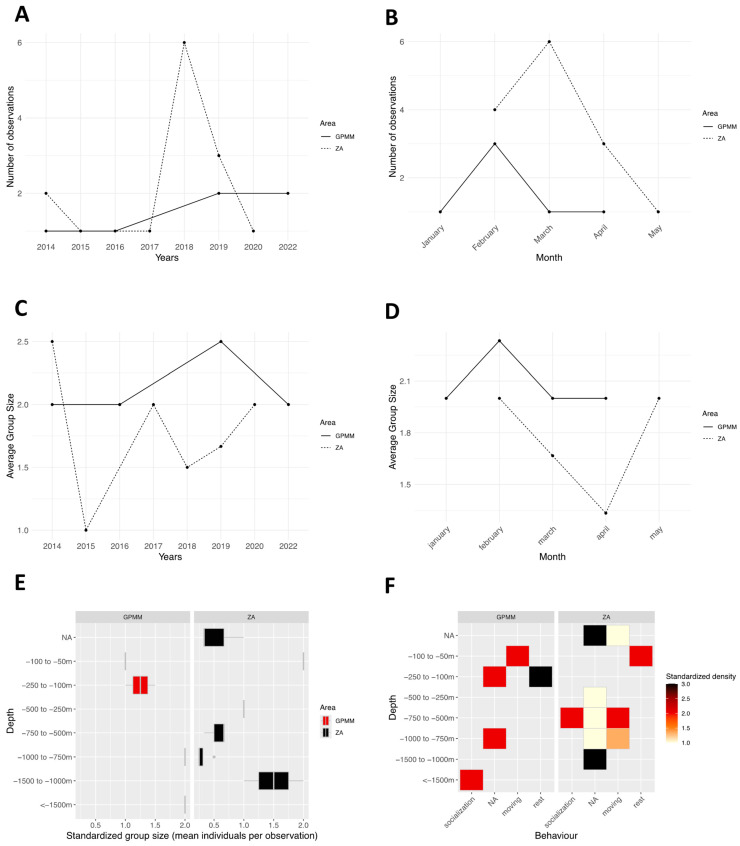
Distribution of *Megaptera novaeangliae* by month (**A**) and year (**B**), the average group size by month (**C**) and year (**D**), the distribution of sightings standardized by depth (**E**), and the density of sightings according to behavior and area by depth (**F**) (ZA: adjacent area; GPMM: Grand Port Maritime de Martinique).

The number of standardized observations by year and study area. Sightings were recorded every year except in 2021. In the GPMM zone, one to two observations were reported annually (Figure 13A). In the adjacent zone, yearly observations ranged from one to six, peaking in 2018. In the GPMM zone, one to three observations were reported monthly from January to April (Figure 13B). In the adjacent zone, one to six sightings were recorded from February to May. Group size remained stable in the GPMM zone, with a minimum of two individuals per observation (Figure 13C). In contrast, group sizes in the adjacent zone ranged from one to two individuals, with heterogeneous variation across years.

Similar trends were observed in the monthly group size patterns (Figure 13D), averaging two individuals per observation in the GPMM zone and fluctuating between one and two individuals in the adjacent zone. Regarding depth distribution (Figure 13E), observations in the GPMM zone were concentrated in shallower waters, primarily between 100 and 150 m, with a few sightings between 50 and 100 m. In the adjacent zone, sightings occurred across a broader depth range, mostly between 1000 and 1500 m, with some observations between 500 and 1000 m. Individual density by behavior and depth (Figure 13F), it was revealed that, in the GPMM zone, socializing individuals were found at depths greater than 1500 m. Traveling behavior was recorded between 50 and 100 m, and resting behavior predominantly occurred between 100 and 250 m. In the adjacent zone, socializing behavior was observed between 500 and 750 m; traveling behavior, between 500 and 1000 m; and resting behavior, between 50 and 100 m.

No foraging behavior was recorded in either study area.

**Figure 14:**
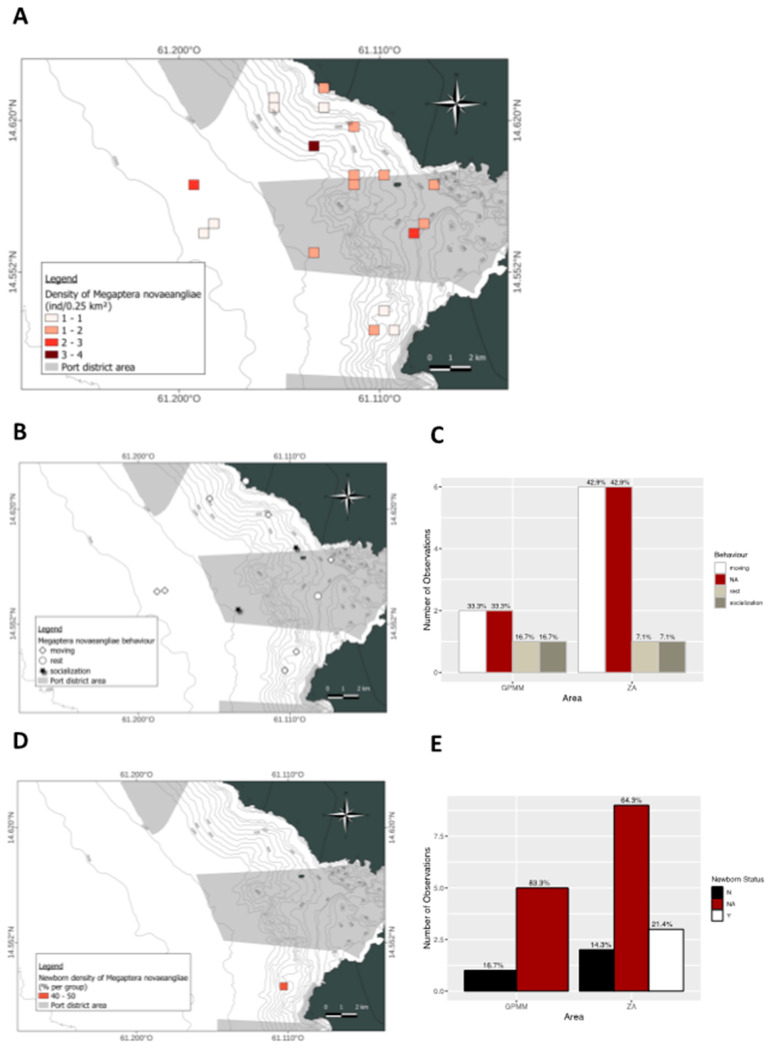
Mapping of *Megaptera novaeangliae*, individual density (**A**), density of juveniles observed (**B**) and distribution by area (**C**), distribution of behavior observed (**D**) and distribution by area (**E**) (ZA: adjacent area; GPMM: Grand Port Maritime de Martinique), (N: any newborn, Y: observation of newborn, NA: no data).

A spatial distribution analysis (Figure 14A) revealed heterogeneous individual distribution. In the GPMM zone, sightings occurred in both the Bay of Fort-de-France and offshore areas. Similarly, in the adjacent zone, individuals were recorded both offshore and near the coast in the northern and southern sections of the study area. Regarding the presence of juveniles (Figure 14B,C), none were observed in the GPMM zone. In contrast, 21.4% of sightings in the adjacent zone included at least one juvenile located in the southern part of the area. Spatial distribution of behaviors in the GPMM zone, traveling was the most frequently observed behavior (33.3%), followed by resting and socializing (16.7% each) (Figure 14D,E). Traveling occurred inside the bay, resting at its entrance, and socializing further offshore. In the adjacent zone, traveling was the most frequently recorded behavior (42.9%), while resting and socializing represented 7.1% of observations each.

## 4. Discussion

An analysis of cetacean observations in the Grand Port Maritime de Martinique (GPMM) and surrounding areas reveals that species use habitats differently, depending on bathymetry, environmental conditions, and human activities. The frequency of sightings allows for the identification of spatiotemporal patterns and provides insights into how cetacean distribution and behavior may vary between areas under different levels of anthropogenic influence, such as the inner bay and offshore zones. Specifically, two coastal species, *Stenella attenuata* and *Tursiops truncatus*, consistently inhabit the area year-round, particularly in shallow waters near port infrastructure, especially at the entrance to and around the bay. For *S. attenuata*, this fidelity to the coastal habitat may reflect specific social or foraging needs, which is consistent with findings in Costa Rica [35], where the species also opportunistically uses the coast. In contrast, *T. truncatus* displays ecological plasticity by exploiting both anthropogenic and natural areas with a stable temporal distribution, especially north of the GPMM. Similar flexible behaviors have been documented in the Mediterranean [36] and Australia [37]. Four species, on the other hand, display a more sporadic presence, likely driven by seasonal or ecological factors: *Lagenodelphis hosei* is primarily observed during the wet season, *Physeter macrocephalus* sporadically appears near the channel, *Globicephala macrorhynchus* rarely sighted occasionally transits in groups through deeper waters, and *Megaptera novaeangliae* frequents the bay from January to May in relation to its seasonal migration [38]. These patterns could not be fully assessed due to dataset limitations. Although the study spans 2013–2022, survey effort varied across years. Data were collected through opportunistic whale-watching operations, primarily during high-tourism periods. This likely led to underrepresentation of offshore or seasonally present species, especially during months with little tourism activity. While seasonal or ecological factors likely influence species occurrence, these trends remain indicative and require confirmation through systematic, year-round surveys.

### 4.1. Groupe Size

Interestingly, group sizes vary markedly by species and zone. *S. attenuata* forms dense groups in the GPMM, suggesting that the area has a social or educational function for juveniles. Conversely, *Tursiops truncatus* is generally observed in small groups in nearshore zones; however, significantly larger groups are recorded at bay exits and shallow coastal areas, likely associated with the presence of calves. In the Bay of Islands, group sizes increased in the presence of young individuals, supporting the hypothesis that calving and social-educational behaviors contribute to higher aggregation nearshore [39]. Substantial variation is also noted for pelagic species: *L. hosei* can form very large groups with significant interannual variation (up to 280 individuals), *G. macrorhynchus* often exceeds 90 individuals, and P. macrocephalus typically appears in small groups of one to three individuals. Slightly larger group sizes are seen in peripheral zones, which may indicate different ecological functions across areas. Bathymetric preferences reveal clear spatial segregation. Coastal species, such as *S. attenuata* and *T. truncatus*, favor shallow areas, particularly in the GPMM. Pelagic species are observed in much deeper waters. *P. macrocephalus* is mostly sighted at depths greater than 1500 m within the port and at depths between 750 and 1500 m at the periphery. G. macrorhynchus uses the deep areas of the GPMM for resting and the peripheral zones for transit. *M. novaeangliae* uses depths between 50 and 150 m for resting and depths between 500 and 750 m for socializing. Additionally, the presence of juveniles in several cetacean groups underscores the ecological significance of the study area as a potential nursery or educational habitat. Social behaviors observed in these groups are often associated with essential learning processes for the development and survival of young individuals. These behaviors are crucial for acquiring skills related to foraging, navigation, and group cohesion. However, the presence of juveniles implies heightened vulnerability, especially in areas subject to anthropogenic pressures. [40,41].

### 4.2. Behaviors

In addition to the presence of juveniles, a behavioral analysis combined with spatiotemporal characteristics highlights distinct habitat use patterns within the GPMM. Grouping species by dominant behavior and recurrence reveals three main behavioral profiles. The first group comprises highly mobile species that use the GPMM in a structured social manner, including the pantropical spotted dolphin (*S. attenuata*) and the bottlenose dolphin (*T. truncatus*). These species exhibit a wide behavioral repertoire, frequently engaging in socializing, traveling, and foraging. This reflects their diverse and intensive use of the habitat. Specifically, *S. attenuata* is observed year-round with a clear preference for depths of less than 100 m, and its large group sizes and intense social interactions suggest strong social structuring and habitat fidelity [42,43].

Interestingly, traveling was the most frequently recorded behavior across all observed species. In the *Stenella* species, the formation of larger groups in certain areas may indicate social cooperation to maximize feeding opportunities, a strategy also observed in other tropical marine environments [44]. Avoidance behaviors observed in areas with high maritime traffic support previous findings on the negative impact of anthropogenic disturbance on cetacean habitat use [15,37]. Similarly, *Tursiops truncatus* exhibits a similar pattern of observation, albeit slightly less frequently. The occurrence of hunting and social behaviors in areas influenced by human activity may reflect a certain degree of behavioral plasticity or tolerance to disturbance. This species is known to adjust its behavior in response to maritime traffic and underwater noise through changes in social organization or activity rhythms [37]. This behavioral flexibility has also been observed in the Indo-Pacific and Florida [45,46]. Therefore, these two species are clearly resident in Fort-de-France Bay, regularly conducting essential activities like feeding, socializing, and traveling. Their strong site fidelity to this area consequently exposes them to significant pressures from maritime traffic and port-related disturbances [46]. The second group comprises pelagic species, such as Fraser’s dolphin and sperm whales, which are primarily observed during foraging or transiting behaviors. These species are rarely seen in the coastal areas of the GPMM, and their appearances are associated with deeper zones [47,48]. Fraser’s dolphins are occasionally sighted, often during foraging and transit, which may reflect temporary exploitation of food resources. Their presence may indicate opportunistic incursions into the bay under favorable conditions or in search of specific prey [47]. The deep-diving sperm whale likely uses the bay’s perimeter as a transit or feeding area along the continental shelf. Though sightings are sporadic, their presence underscores the ecological importance of the port’s peripheral zones, where complex trophic interactions may occur [49]. While these species do not appear to be residents, their repeated presence suggests they use the habitat functionally, primarily for feeding. The third group consists of pelagic species that temporarily use the bay, such as short-finned pilot whales and humpback whales. These species use the GPMM seasonally, typically for migration or social gatherings. Though pilot whales are infrequently observed, they are always seen in groups displaying social behaviors, possibly indicating phases of rest or social interaction during their movements throughout the wider Caribbean [50]. Humpback whales, which are highly mobile, use the bay mainly during the breeding season. The presence of adults and mother-calf pairs supports the hypothesis that the habitat is temporarily used for reproduction [51,52].

However, this trend must be interpreted with caution due to potential observer bias. The data were primarily collected during whale-watching excursions, during which moving groups are more easily detected and followed. This may result in an underrepresentation of stationary behaviors, such as resting or socializing. Nevertheless, traveling remains a key activity that reflects how cetaceans use the area. It may also indicate the need for undisturbed movement corridors within the study region and functional connectivity between zones. These findings underscore the importance of maintaining habitat quality for non-resident species as well. This classification underscores the importance of Fort-de-France Bay and the Grand Port Maritime of Martinique (GPMM) as multifunctional areas for cetaceans while highlighting the risks posed by human disturbances [43,53].

### 4.3. Influence of Marine Traffic

However, Fort-de-France Bay faces significant threats to cetaceans and their behavior. Intense maritime traffic poses a significant threat, especially to resting dolphins and migrating whales. Ship strikes are a constant danger, especially during whale migration or dolphin resting phases [22,54]. All cetacean species are vulnerable to collisions, but large, slower-moving species, such as sperm and humpback whales, are especially at risk because they spend more time at the surface [55,56]. In the study area, small cetaceans such as pantropical spotted dolphins, Fraser’s dolphins, and bottlenose dolphins are potentially at risk of ship strikes due to the overlap between their habitat use and intense maritime traffic [57]. Underwater noise from maritime traffic exacerbates this issue further. This noise masks cetacean vocalizations and communication signals [40], which can disrupt group cohesion and social organization [58]. These behavioral changes and chronic stress may ultimately affect individual health and population viability over time [26]. Notably, known ferry routes in the study area overlap with zones where essential behaviors, such as resting and foraging, are observed. Frequent vessel transits in these areas likely disrupt these essential behaviors, affecting feeding efficiency and recovery during rest periods. Previous research in Martinique has shown that underwater noise negatively impacts dolphin communication [52], suggesting that reduced socialization behaviors and fewer groups with juveniles occur in heavily disturbed areas. In addition to commercial traffic, whale-watching activities around the island also target the species observed in the bay. Despite regulations and guidelines for proper approach distances [59], rule violations remain common [60]. Even occasional violations can disturb cetaceans in both the short and long term. A study of humpback whales in Alaska highlighted the potential long-term behavioral effects of whale watching [61]. Similar effects have been reported on the communication and whistle patterns of pantropical spotted dolphins in Martinique [53]. These factors contribute to the multiple, concurrent pressures faced by Fort-de-France Bay and its surroundings. Despite management plans and conservation measures, persistent threats and regulatory lapses may continue to impact cetacean behavior and habitat use in the long term. This underscores the importance of ongoing research and monitoring efforts to assess these impacts and refine conservation strategies accordingly.

## 5. Conclusions

This study sheds new light on the use of the Bay of Fort-de-France by six cetacean species, revealing a fine-scale, spatiotemporal structuring of their distribution linked to factors such as depth, geographical zones, and behavioral states. Analyses revealed the regular presence of certain species in areas with high anthropogenic pressure, especially within the Grand Port Maritime de Martinique. Using these zones for critical behaviors such as resting or socializing raises important conservation concerns.

These findings confirm the bay’s and its periphery’s biological importance as multifunctional habitats that support foraging, resting, and social interactions. The first step toward mitigation is the identification and mapping of key ecological zones, which could guide future protective measures. Management recommendations could include implementing reduced vessel speeds to lower noise and collision risks, as well as defining navigation corridors for recreational boats to limit disturbances in sensitive areas. Ongoing research, such as passive acoustic monitoring programs, contributes to a deeper understanding of the bay’s soundscape and the acoustic behavior of cetaceans, particularly elusive or nocturnal species. Future conservation strategies could benefit from integrating these acoustic insights with ethological observations alongside awareness-raising initiatives for maritime personnel. This integrated approach is crucial for balancing port development with the protection of marine biodiversity, aligning with regional needs and international conservation frameworks.

## Figures and Tables

**Table 1:** Behavioral categories of cetaceans with observation criteria and examples.

Behavioral Examples	Observation Criteria	Behaviors
Long and repeated dives, temporary group dispersion followed by regrouping, active surfacing, seabirds diving nearby.	Erratic or circular movements, prolonged dives, coordination among individuals, presence of seabirds or visible prey.	Foraging
Parallel alignment of individuals, prolonged floating, slow or stationary movement.	Compact formation, low speed, steady direction, regular and spaced breathing, limited social interaction.	Resting
Synchronized swimming, leaping, tail slapping, playing at the boat’s bow, rolling	Frequent physical contact (rubbing, overlapping), vocalizations, playful behaviors (e.g., leaps, rolls, bow-riding)	Socialization
Straight-line movement, steady group travel without clear signs of other activities.	Linear or slightly dispersed formation, constant speed, steady direction, limited interaction among individuals.	Traveling

## Data Availability

The whole dataset supporting the findings of this study is available on demand (b.montgolfier@aquasearch.fr).
